# Identification of heterotic loci associated with grain yield and its components using two CSSL test populations in maize

**DOI:** 10.1038/srep38205

**Published:** 2016-12-05

**Authors:** Hongqiu Wang, Xiangge Zhang, Huili Yang, Xiaoyang Liu, Huimin Li, Liang Yuan, Weihua Li, Zhiyuan Fu, Jihua Tang, Dingming Kang

**Affiliations:** 1 College of Agriculture and Biotechnology, Beijing Key Laboratory of Crop Genetic Improvement, China Agricultural University, Beijing, 100193, China; 2 Key Laboratory of Wheat and Maize Crops Science, Collaborative Innovation Center of Henan Grain Crops, College of Agronomy, Henan Agricultural University, Zhengzhou, 450002, China; 3 Hubei Collaborative Innovation Center for Grain Industry, Yangtze University, Jingzhou, 434023, China

## Abstract

Heterosis has widely been used to increase grain yield and quality. In this study, the genetic basis of heterosis on grain yield and its main components in maize were examined over 2 years in two locations in two test populations constructed from a set of 184 chromosome segment substitution lines (CSSLs) and two inbred lines (Zheng58 and Xun9058). Of the 169 heterotic loci (HL) associated with grain yield and its five components identified in CSSL × Zheng58 and CSSL × Xun9058 test populations, only 25 HL were detected in both populations. The comparison of quantitative trait loci (QTLs) detected in the CSSL population with HL detected in the two test populations revealed that only 15.46% and 17.35% of the HL in the given populations respectively, shared the same chromosomal regions as that of the corresponding QTLs and showed dominant effects as well as pleiotropism with additive and dominant effects. In addition, most of the HL (74.23% and 74.49%) had overdominant effects. These results suggest that overdominance is the main contributor to the effects of heterosis on grain yield and its components in maize, and different HL are associated with heterosis for different traits in different hybrids.

The heterozygous F_1_ generation often exhibits better performance than its homozygous parents, a phenomenon known as heterosis or hybrid vigour^1^,^2^. Heterosis plays an important role in the improvement of crop productivity, nutrient quality and resistance to biotic and abiotic environmental stresses^3^,^4^. The development of heterotic crops, particularly hybrid rice and maize, is one of the most important applications of genetics in agriculture. Currently, over half of global rice and maize production is from hybrid seeds, which have resulted in tremendous increases in yield^5^,^6^. In classical genetics, three main hypotheses have been proposed to explain the genetic basis of heterosis: dominance, overdominance, and epistasis^7^. The dominance hypothesis emphasizes the masking of deleterious recessive alleles between parents in the hybrid^8^,^9^. In rice, quantitative trait loci (QTLs) analysis in an indica–japonica recombinant inbred line (RIL) backcross population has suggested that dominance complementation is the major cause of heterosis^10^. The overdominance hypothesis attributes heterosis to the superiority of heterozygotes over parental homozygotes at individual loci^9^,^11^. Such single-locus overdominance of heterozygous alleles has shown to result in heterosis directly in rice^3^, Arabidopsis^12^, tomatoes^13^, and maize^14^. According to the epistasis hypothesis, positive epistatic interactions between non-allelic genes are responsible for heterosis^15^,^16^. For example, Yu *et al*.^17^ have detected a large number of digenic interactions associated with yield and its component traits in hybrid rice in an F_2:3_ population. In addition, epistasis has been revealed to contribute significantly to the heterosis of growth-related traits in Arabidopsis^18^,^19^,^20^. Various phenomena including hormonal regulation and metabolism^21^,^22^,^23^, genomic structural variations^24^,^25^, changes in global expression trends^26^,^27^,^28^, regulation of small RNAs^29^,^30^, post-transcriptional modifications^31^,^32^,^33^ and epigenetic effects^34^,^35^ have recently been associated with heterosis of specific organs and developmental stages at the molecular level. In addition, the effects of various genes^36^,^37^,^38^,^39^ and gene dosages on heterosis^40^,^41^,^42^ have been reported in previous studies. Although the above studies have suggested that heterosis arises from a complex genetic basis and multi-level molecular mechanism, yet the genetic basis of heterosis remains unclear.

To reveal the genetic basis of heterosis, the use of appropriate experimental designs and materials is critical. Early research on heterosis primarily used different F_2_ and backcross populations^16^,^43^. Subsequently, diallelic and extended design III (triple test cross) populations were also applied in combination with genome-wide genotyping data to dissect the genetic basis of heterosis^16^. More recently, a novel informative approach involving “immortalized F_2_” (IF_2_) populations has been developed for heterosis research in rice^3^,^44^,^45^. Unfortunately, all of the above-mentioned populations suffer from a common problem: their complex genetic background. Compared with other mapping populations, chromosome segment substitution lines (CSSLs) have a simple genetic background, with the exception of one or a few homozygous chromosome segments from the donor parent. CSSLs have been used to study heterosis in rice^46^ and tomatoes^47^. Using testcross hybrids developed from 140 introgression line populations from two parental accessions, Meyer *et al*.^48^ have reported a QTL for early stage heterosis for biomass in Arabidopsis. Recently, 15 QTLs that are also HL contributing to heterosis regarding plant height acting dominantly have been detected in a CSSL population and its corresponding test population in rice^49^.

Grain yield, a complicated trait that comprises several major components in different crops, is affected by many genetic and non-genetic factors. In rice, HL associated with yield and its components have been detected in hybrid populations derived from crosses between CSSLs and their recipient/donor parents^50^. Tang *et al*.^51^ have reported that dominance effects of HL at the single-locus level as well as AD interactions play an important role in the genetic basis of heterosis for grain yield and its components in the maize hybrid Yuyu22. Wei *et al*.^52^ have found that dominance and overdominance are two important components of heterosis in maize grain yield and yield-related traits. However, genetic analysis of heterosis in maize always depends on a segregated population derived from two parents and therefore do not permit the comparison of the genetic effects of a single HL between different parents. In the present study, HL associated with grain yield and its major components were studied in two test populations constructed from a CSSL population and two test inbred lines through comparison of each single test cross with its corresponding hybrid (CK). The objectives of this study were therefore (1) to detect the HL underlying grain yield and its components, (2) to compare the identified HL associated with grain yield and its components between different test populations, and (3) to analyse the genetic basis of heterosis for grain yield and its components in maize.

## Results

### Grain yield and its main components in the test populations

The current study focused on a population of 184 maize CSSLs constructed from the elite inbred lines lx9801 and Chang7-2. The two inbred lines were derived from the Tangsipingtou maize heterosis group in China, and the test parents, Zheng58 and Xun9058, were derived from the corresponding modified Reid heterosis groups.

The ear length in the CSSL population ranged between 8.64–15.85 cm within an average of 12.04 cm. The mean value of this trait in the recipient parent lx9801 was slightly higher than that in CSSL population (Table 1). The mean ear width in the CSSL population was 4.16 cm, which was lower than the mean in the recipient parent lx9801; the same trend was true for row number, kernels per row, and 100-kernel weight. However, the mean grain yield in the CSSL population was 6.24 t ha^−1^, which was higher than that of lx9801.

To detect the HL of grain yield and its main components in the two test populations, the corresponding hybrids, lx9801 × Zheng58 or lx9801 × Xun9058, were used as the CK. The average grain yield of the Zheng58 × lx9801 hybrid was 11.19 t ha^−1^ in the four environments (two locations for 2 years), with a mid-parent heterosis of 72.02% (Table 1). In the CSSL × Zheng58 population, the mean grain yield recorded in the four environments was 11.05 t ha^−1^, within a range of 8.91–12.76 t ha^−1^ with an average mid-parent heterosis of 69.87%. The mean value for kernels per row in the given test population was 34.44 within the range of 31.23–36.92, with 49.77% mid-parent heterosis. Average mid-parent heterosis in the test population for the other four measured traits was as follows: ear length (36.72%), 100-kernel weight (14.45%), ear width (13.66%), and row number (7.62%). In addition, the average mid-parent heterosis of the test population was almost equal to that of the hybrid Zheng58 × lx9801.

In the CSSL × Xun9058 population, large variations in grain yield and its five components were observed in the four environments (Table 1). The mean grain yield of this test population, 10.97 t ha^−1^, showed substantial variation (9.38–12.35 t ha^−1^) across the four environments. The mid-parent heterosis for this trait was 62.92%. The trait with the second highest mid-parent heterosis was kernels per row, with a mean value of 34.15 and 44.93% mid-parent heterosis in the four environments. For the other measured traits in the test population, the average mid-parent heterosis values from highest to lowest were 30.52% (ear length), 16.90% (100-kernel weight), 12.32% (ear width), and 8.61% (row number).

According to combined analysis of variance, the six measured traits exhibited significant variations in locations and genotypes at *p* < 0.05 and *p* < 0.01 levels (Table 2). However, only ear length showed significant variation in location × genetic effects at the *p* < 0.05 level. The heritability (*H*^*2*^_*B*_) values of ear length, ear width, row number, kernels per row, 100-kernel weight, and grain yield were 63.02%, 67.26%, 68.06%, 62.53%, 62.08% and 73.28% respectively.

### Detected QTLs associated with grain yield and its main components in the CSSL population

A QTL was considered to exist in the CSSL population when a significant difference was observed in the measured value of a trait between the CSSL and the recurrent inbred line lx9801 (*p* < 0.05). Six QTLs associated with ear length were identified based on the average value of each CSSL in the four different environments (Table 3). Among them, QTL *qEL1a*, located in bin 1.03, had a −12.26% contribution to phenotypic variation and decreased the average ear length by 1.49 cm. The second QTL was *qEL9*, which accounted for −11.44% of the average phenotypic variation in the four environments, with a −1.39 cm additive effect. Of the nine detected QTLs associated with ear width located on chromosomes 1, 4, 5, 6, and 9, only one (*qED5*) had a positive contribution in the four environments. Eight QTLs associated with row number were identified: three (*qRN3, qRN5*, and *qRN9*) with positive additive effects and five (*qRN2, qRN4, qRN6a, qRN6b*, and *qRN6c*) with negative additive effects. Nine QTLs associated with kernels per row were identified in the four environments. The QTL *qKPR3* had a 16.46% average phenotypic contribution to kernels per row, whereas a second QTL, *qKPR1a*, had a −14.86% average phenotypic contribution in the CSSL population. Another major QTL, *qKPR1b,* had an 11.08% average contribution.

Of the seven QTLs identified to be associated with 100-kernel weight, QTL *qKW2*, with a 3.12 g additive effect, had the highest contribution in the CSSL population. The second most influential QTL was *qKW1a*, which had a −11.47% phenotypic contribution to 100-kernel weight. Of the six detected QTLs associated with grain yield, QTL *qGY1* explained −20.02% of the average phenotypic variation in the four environments. The second highest-contributing QTL associated with grain yield was *qGY2*, which accounted for 16.86% of the phenotypic variation.

### Identified HL associated with grain yield and its components in the two test populations

HL associated with the measured trait were considered to exist in the chromosomal region of the receptor parent and donor parent as well as the test parent when the value of the measured trait in the single test hybrid differed significantly from that of its corresponding hybrid. Twenty-nine different HL associated with ear length were identified in the two test populations, including 16 and 17 HL in the CSSL × Zheng58 and CSSL × Xun9058 populations, respectively (Tables 4 and 5). The majority of HL (25; 86.21%) were detected in only one test population. Among the HL detected in both test populations, the HL *hEL7e* had −6.90% and −7.73% contributions to over-standard heterosis for ear length in the Zheng58 and Xun9058 test populations, respectively, whereas HL *hEL1b* had corresponding values of −4.72% and 6.04%. The third HL detected in both test populations, *hEL6d*, was responsible for 8.00% and −1.91% of over-standard heterosis, and the HL *hlEL2b* contributed −3.84% and −1.94% over-standard heterosis for ear length in the two test populations (Table 6).

Of the 29 different HL associated with ear width identified across the four environments, only four HL were detected in both test populations. The HL *hlEW1c*, located on chromosomal bin 1.05, had contributions of −4.74% and −2.53% to over-standard heterosis associated with ear width in the Zheng58 and Xun9058 test populations, respectively. Another HL, *hlEW6a*, which is located on chromosomal bin 6.00 between simple sequence repeat (SSR) markers phi075 and umc2309, accounted for −4.14% and −4.17% of over-standard heterosis for ear width, respectively. The other two HL identified in both populations were *hlEW6b* and *hlEW6c*, which had contributions to over-standard heterosis for ear width of −3.08% and −2.90% in the Zheng58 test population, with corresponding values of −8.83% and −4.37% in the Xun9058 population.

We detected 25 different HL associated with row number, of which five were identified in both test populations. One HL, *hRN1a*, was located in chromosomal bin 1.04 between SSR markers bnlg182 and umc1144; it accounted for 5.88% and 7.92% of over-standard heterosis for row number in the Zheng58 and Xun9058 test populations, respectively. The HL *hRN4* had −4.15% and 8.39% phenotypic contributions to over-standard heterosis for row number in the Zheng58 and Xun9058 test populations, respectively. The HL *hRN9a, hRN9e*, and *hRN10* were also detected in both test populations.

Out of the 30 different identified HL associated with kernels per row, three were identified in both test populations. The HL *hKPR1a*, located on chromosomal bin 1.02, had −10.24% and 8.41% contributions to over-standard heterosis for kernels per row in the Zheng58 and Xun9058 test populations, respectively. Another HL, *hKPR2a*, had −11.54% and 7.95% contributions to over-standard heterosis for kernels per row in the Zheng58 and Xun9058 test populations, respectively. In addition, the HL *hKPR7a* accounted for 12.99% and 8.53% of over-standard heterosis for kernels per row in the two test populations.

Among the 30 different HL associated with 100-kernel weight identified in the two test populations, only four HL were detected in both test populations. The HL *hKW7a* had 15.82% and −12.69% contributions to over-standard heterosis for 100-kernel weight in the Zheng58 and Xun9058 test populations, respectively. Another HL, *hKW9a*, had −10.37% and −12.60% phenotypic contributions to over-standard heterosis for 100-kernel weight in the two test populations, respectively. HL *hKW6g* and *hKW9b* were also detected in both test populations.

We detected 26 HL associated with grain yield in the two test populations. The HL *hGY1d*, which was identified in both test populations, had a high contribution to over-standard heterosis for grain yield (11.04% and 11.42% in the Zheng58 and Xun9058 test populations, respectively). The HL *hGY6c*, which had contributions of −8.98% and 18.00% to over-standard heterosis for grain yield in the Zheng58 and Xun9058 test populations, respectively, was located in chromosomal bin 3.03. Two other HL, *hGY1a* and *hGY3a*, were also detected in both test populations.

### Overdominant effects play an important role in heterosis for grain yield and its components

Theoretically, if an HL or QTL is identified in both a test hybrid and its corresponding CSSL, it should exhibit a dominant effect; in contrast, if the HL is identified in only a particular test hybrid with no corresponding QTL in the associated CSSL, it should have an overdominant effect. A comparison between the QTLs detected in the CSSL population and the HL in the two test populations revealed that only 15.46% (15/97) and 17.35% (17/98) of the HL identified in the Zheng58 and Xun9058 test populations, respectively, had corresponding QTLs in the CSSL population. These HL would be expected to show dominant effects; in contrast, the remaining HL (84.54% and 82.65%) associated with grain yield and its five components in the Zheng58 and Xun9058 test populations, which did not have corresponding QTLs in the CSSL population, should act in an overdominant manner in the two test populations. These results suggest that overdominant effects play an important role in heterosis for grain yield and its components in maize.

### Confirmation of the two major HL, *hlEW2b* and *hlEL3d*, in a sub-CSSL test population

In this study, 14 sub-CSSL test hybrids were constructed by crossing CSSLs bearing the HL *hlEW2b* with the test parent Zheng58. Of these test hybrids, three sub-CSSL test hybrids that possessed the donor chromosomal region between SSR markers bnlg1064 and umc1024 exhibited significant differences in ear width compared with that in the lx9801 × Zheng58 hybrid at both the Xunxian and Changge locations in 2014 (Supplementary Table 1 and Supplementary Figure 1).

We also generated 17 sub-CSSL test hybrids derived from CSSLs harbouring the HL *hlEL3d* crossed with inbred line Zheng58. Five of the resulting sub-CSSL test hybrids, which included the donor chromosomal region between the SSR markers umc1489 and umc1825, displayed significant differences in ear length compared with the lx9801 × Zheng58 hybrid at both the Xishuangbanna and Sanya locations in the winter of 2015 (Supplementary Table 2 and Supplementary Figure 2).

## Discussion

Because quantitative trait phenotypes reflect both additive and dominant gene effects, the acquisition of accurate performance data for heterosis for a measured trait is difficult. Consequently, mid-parent heterosis data have often been used to detect HL or to estimate the dominant effect of QTLs. Among the different types of segregated populations used to dissect the genetic basis of heterosis, such as F_2_, doubled-haploid, recombinant inbred lines, IF_2_ and triple testcross populations^17^,^43^,^45^,^53^, IF_2_ populations are considered to be ideal because they can identify HL and digenic interactions directly on the basis of mid-parent heterosis^45^. Despite this advantage, HL and digenic interactions identified in an IF_2_ population still exist in the complicated genetic background population. CSSL populations backcrossed with the recipient parent have been widely used to identify HL in crops such as rice^46^,^49^,^50^, tomatoes^47^ and cotton^4^, but cannot detect the digenic interaction of heterosis. In this study, HL associated with grain yield and its components were identified by comparing CSSL test hybrids to their corresponding CK in two test populations. Because the test parents were derived from the corresponding heterotic groups, each CSSL test hybrid should have whole-genomic heterozygous loci. Consequently, the detected HL used in the test population include two types of interactions: HL at the single-locus level and digenic interactions at the two-locus level.

In previous studies, heterotic QTLs (hQTLs) or HL have usually been detected in a set of test or backcross populations^47^,^54^,^55^; however, the different studies have rarely used identical or similar genetic backgrounds, thus making it difficult to compare the HL or hQTLs identified in different populations. In this study, two test populations constructed from a CSSL population and two inbred lines were used to identify the HL associated with grain yield and its five components in maize. Importantly, the two test inbred lines, Zheng58 and Xun9058, belong to the same major heterotic group, that of Reid germplasm. In a comparison of the detected HL associated with grain yield and its components in the two test populations, only 25 (25.77% and 25.51%) HL were detected in both the Zheng58 and Xun9058 test populations. In fact, most HL (72/97, 74.23%; 73/98, 74.49%) identified in the Zheng58 and Xun9058 test populations were different, thus supporting the hypothesis that heterosis is generally the result of the action of multiple loci, with different loci affecting heterosis for different traits in different hybrids^56^.

Dominance and overdominance are the two main hypotheses used to explain the genetic basis of heterosis. One of the most direct approaches to document the relative roles of dominance and overdominance is analysis of hQTLs. In rice, dominance or overdominance and epistasis are believed to play an important role in yield-related traits^57^,^58^, but the relative importance of these three phenomena is under debate. For example, Tang *et al*.^51^ have found that the dominance effect of HL at the single-locus level plays an important role in grain yield and its components in the hybrid maize Yuyu22. In contrast, Guo *et al*.^4^ have identified three genetic effects (partial dominance, full dominance, and overdominance) on yield and other agronomic traits in cotton, with the overdominant effect having the highest contribution to heterosis. Shen *et al*.^49^ have reported that dominance is the main contributor to heterosis for plant height in rice. Semel *et al*.^47^ have conducted a detailed analysis of heterosis in tomatoes and have provided evidence for higher levels of overdominant action for traits associated with reproductive fitness. Huang *et al*.^54^ have reported that the accumulation of numerous rare superior alleles with positive dominance is an important contributor to the heterotic phenomenon in rice. Finally, Wang *et al*.^59^ have observed that the heterozygous alleles of pentatricopeptide repeat proteins (*RsRf3-1*/*RsRf3-2*) restore male fertility, an expressed overdominant effect, to cytoplasmic male-sterile radishes.

Theoretically, the QTLs detected in the CSSL population may have two genetic effects: additive and simultaneous additive and dominance/overdominance. The HL detected in the test population should have a dominance or overdominance effect. When the QTL and HL are detected in the CSSL population and its test population simultaneously, the QTL or HL should have an additive and dominance/overdominance effect, which is pleiotropism. Additionally, the dominance and overdominance analyses in the previous study primarily depend on the ratio of the dominant effect to the additive effect for one QTL or HL. However, some QTLs or HL may have only a dominant or an additive effect. For example, the majority of detected HL associated with grain yield and its components in this study had no consistent QTLs and this type of HL should have an overdominant effect. However, some detected HL associated with grain yield and its components in the two test populations had consistent QTLs in the CSSL population, according to classical genetics, the HL should show a dominant effect. Nonetheless, the HL were identified in a long chromosomal region that may have included several different HL; consequently, the observed effect of the HL may have been pseudo-overdominance. Nevertheless, 84.54% and 82.65% of HL expressed overdominant effects in the two test populations (Table 6). Although several HL may have exerted pseudo-overdominant effects, most of the detected HL associated with grain yield and its main components exhibited overdominant expression. Therefore, in the test population, overdominance plays an important role in heterosis for grain yield and its main components at the single-locus level in maize^52^.

Previous studies detecting HL have always used two types of segregated populations, the IF_2_ population or CSSL backcross population^3^,^4^,^47^,^50^, and the effect of HL identified in the populations existed in only a pair of alleles between two parents. Therefore, the HL effect between different parents could not be analysed. In fact, the common HL between different parents may have various effects and show different heterotic values. In this study, the HL were detected through comparison of significantly different measured traits between a single hybrid in the test population to its corresponding hybrid: HL% = (H − CK)/CK × 100%, and the value of over-standard heterosis for HL may be positive or negative. When a common HL was detected in two test populations, owing to its having various effects between two different pairs of parents, the HL sometimes showed opposite values in the two test populations. In fact, out of the 25 detected common HL associated with grain yield and its components in this study, 48.00% (12/25) had a positive value in one population and a negative value in the other population, and only 52.00% (13/25) had a consistent effect (Table 6), thus implying that the HL had various effects between different parents.

Given the genetic effects of additive genes and HL associated with quantitative traits superimposed in a single hybrid, an ideal strategy for distinguishing QTL and HL effects is the use of different segregating populations. In a previous study using a chromosome segment introgression line population in cotton, Guo *et al*.^4^ have reported that only 12.08% of HL (7/58) were also detected by QTL analysis. Tang *et al*.^60^ have found that 25% of QTLs and 30% of HL associated with plant height in an IF_2_ population in maize had the same chromosomal locus. In another study in maize, Wei *et al*.^61^ have determined that only 27.03% of HL associated with five morphological traits were located in the same position as a corresponding QTL (24.39%). Comparison of QTLs detected in the CSSL population and HL detected in the two test populations in our study revealed that only 16.49% (16/97) and 15.31% (15/98) of the HL identified in the Zheng58 and Xun9058 test populations were also detected in the CSSL population. Extending the results of QTL and HL analyses in previous studies, we also found that phenotypic traits and heterosis are controlled by two different genetic and molecular mechanisms.

Identification of high-performing hybrids is an integral part of every maize breeding programme. Because field evaluation of all potential hybrids is resource intensive, only a small subset can actually be tested in field trials^62^, and only a few elite hybrids can be selected. Prediction of hybrid performance is thus a very important element of maize breeding^63^. Recent studies have used molecular markers and QTLs associated with genomic prediction of hybrid performance in maize^64^,^65^,^66^, sunflowers^67^, and wheat^68^. One important component of hybrid performance is the specific combination ability between parental lines of a hybrid. Dominance effects of markers must therefore be estimated in addition to additive effects to account for the entire genetic variance. A further complication is that parental lines in hybrid breeding are taken from genetically distant populations to maximize heterosis^62^. Identification of HL associated with important agricultural traits between heterotic patterns is consequently vital for hybrid performance prediction in maize breeding. For optimal exploitation of heterosis, the parental inbred lines of maize hybrids are taken from genetically distant pools of germplasm, called heterotic groups^69^, and have been widely used by maize breeders. In China, Tangsipingtou and modified Reid are the first heterotic groups, which have been widely used in maize breeding^70^. In this study, two test populations, constructed with representative inbred lines derived from the Tangsipingtou and Reid heterotic groups were used to detect HL associated with grain yield and its components in maize. We detected 23 HL that were consistent across the two test populations. These HL associated with grain yield and its components and their associated molecular markers may be used to predict hybrid performance in future maize breeding experiments.

## Methods

### Construction of CSSL and test populations

A population of 184 maize CSSLs constructed from two elite inbred lines, lx9801 and Chang7-2, was used in this study. These two elite inbred lines belonged to the Tangsipingtou heterotic group, an important local germplasm widely used in China. Chang7-2, used as the donor parent, is one parent of the elite hybrid Zhengdan958 and the first commercial hybrid used widely in China (from 2005 to 2015). The recipient parent, lx9801, is a parent of Ludan9002, another elite commercial hybrid. The other (female) parent of both Zhengdan958 and Ludan9002 is Zheng58. We used 225 SSR markers from the IBM 2008 Neighbors maize linkage map (http://www.maizegdb.org/data_center/map) that were polymorphic between the two inbred lines to construct the CSSL population.

The total length of the 184 generated CSSL fragments was 1683.33 cM, with an average length of 9.25 cM, corresponding to 35.5% coverage of the maize genome. The breakdown of SSR fragment sizes and frequencies was as follows: 0.09–69.20 cM (119), 0.01–10.00 cM (82; 68.91% of total CSSLs), 10.01–20.00 cM (31; 26.05%), and >50.00 cM (2; 1.68%)^71^.

Two test populations were constructed using the 184-CSSL population and two inbred lines, Zheng58 and Xun9058. These two inbred lines belong to the improved Reid heterotic group (NBSSS), which is derived from the heterotic model hybrid Reid × Tangsipingtou and broadly used in China. The CSSLs population and the two test inbred lines were planted in the winter of 2011 and 2012 in Sanya (China, N18°15′, S109°30′). Half of the plants from the CSSLs population were used as female parents and manually crossed with two test inbred lines, and the others were selfed at the same time in the field each year.

### Field experiments

The two test populations and their corresponding hybrids (Zheng58 × lx9081 and Xun9058 × lx9081) were evaluated on the farms of the Hebi Agricultural Institute at Xunxian (E 114° 33′, N 35° 41′) and Changge (E 113° 29′, N 34° 1′). Plants were planted after the wheat harvest on the 15^th^–20^th^ of June 2012 and 2013. The experimental design consisted of a randomized complete block design with three replicates; the corresponding hybrids (Zheng58 × lx9801 or Xun9058 × lx9801) were added as controls between every 10-test crosses. Each plant material occupied one plot in the field. Rows in each plot were 4 m long, with 0.66 m spacing between rows. The population density was 67,500 plants ha^−1^. To analyse QTL effects, the CSSL population and the three inbred lines (lx9801, Zheng58, and Xun9058) were planted in the same field according to the same experimental design, and the inbred line Chang7-2 was added as a control in the field. The field was managed according to local maize cultivation practices.

### Performance measurement

After maturity, 10 ears from consecutive plants in each plot were harvested and air dried to a grain moisture level of 13%. The following traits were measured: grain yield (t ha^−1^), ear length (cm), ear width (cm), row number, kernels per row, and 100-kernel weight (g). All traits except grain yield were measured on individual ears. The average value of each test hybrid or CSSL in the four environments was then calculated for further HL or QTL mapping.

### Data analysis

The mid-parent heterosis (H_MP_) of six measured traits in the two test populations was evaluated using the average data from the four environments. Mid-parent heterosis values were calculated as H_MP_ (%) = (F_1_ − MP)/MP × 100% 45, where H_MP_ is the percentage of mid-parent heterosis, F_1_ is the average data of six measured traits in each hybrid in the two test populations over the four environments, and MP refers to the mean of the average values of each CSSL and the corresponding test parent in the four environments. Mid-parent heterosis values of the corresponding hybrids (lx9801 × Zheng58 and lx9801 × Xun9058) were also calculated using the same formula.

One-way analysis of variance (ANOVA) and Duncan’s multiple comparisons were conducted using SPSS 17.0 software. A QTL was considered to exist in the CSSL population when a significant difference was observed in the measured value of a trait between the CSSL and the recurrent inbred line lx9801 (*p* < 0.05). The QTL additive effect was calculated using the following equation: A = (CSSL − lx9801)/2, where A is the additive effect, and CSSL and lx9801 refer to the measured value for a given trait in the two respective lines. The contribution of phenotypic variation (A%) was then calculated as follows: A% = (CSSL − lx9801)/lx9801 × 100%.

HL associated with one of the six measured traits were considered to exist in the test inbred line in the chromosomal region corresponding to the region between the receptor parent and donor parent when the value of the measured trait in the single test hybrid (T_1_ or T_2_) differed significantly from that of its corresponding hybrid, lx9801 × Zheng58 (CK_1_; *p* < 0.05) or lx9801 × Xun9058 (CK_2_; *p* < 0.05), according to one-way ANOVA and Duncan’s multiple comparisons^61^. The over-standard heterosis effect was calculated as follows: HL% = (H_1_ − CK_1_)/CK_1_ × 100%, or (H_2_ − CK_2_)/CK_2_ × 100%, where H_1_ and H_2_ refer to the values of the trait of the single cross in the CSSL × Zheng 58 and CSSL × Xun9058 populations, and CK_1_ and CK_2_ are the values of the trait for the hybrids lx9801 × Zheng 58 and lx9801 × Xun9058, respectively^46^.

As a consequence of the experimental design used for the CSSLs and the two test populations, QTLs detected in the CSSL population should have additive effects. The HL detected in the test populations should express a dominant or overdominant effect. If an HL and a QTL were identified in both a test hybrid and its corresponding CSSL, the HL should theoretically exhibit a dominant effect, because the CSSL population would have a single different chromosomal section compared with that of the recurrent parent. If the HL was identified in only a particular test hybrid with no corresponding QTL in the associated CSSL, however, the HL would be expected to have an overdominant effect.

### Confirmation of two HL associated with ear length and ear width in sub-CSSL test populations

To further verify the HL detected in the CSSL × Zheng58 test population, two CSSLs, 10su076-3 and 10su087-3 carrying HL *hlEL3d* and *hlEW2b* associated with ear length and ear width, respectively, were crossed with the recurrent parent lx9801 to construct a sub-CSSL population. Linked molecular markers were then used to select sub-CSSLs with different homozygous chromosomal sections. Fourteen and seventeen sub-CSSLs derived from the two CSSLs were crossed with the inbred line Zheng58. The sub-CSSL test hybrids containing *hlEW2b* were evaluated at Xunxian (E114°33′, N35°41′) and Changge (E113°29′, N34°1′) in Henan Province in 2014, whereas the sub-CSSL test hybrids harbouring *hlEL3d* were evaluated at Xishuangbanna, Yunan Province (E100°47′, N22°0′) and Sanya, Hainan Province (E109°31′, N18°14′) in the winter of 2015.

## Additional Information

**How to cite this article**: Wang, H. *et al*. Identification of heterotic loci associated with grain yield and its components using two CSSL test populations in maize. *Sci. Rep.* **6**, 38205; doi: 10.1038/srep38205 (2016).

**Publisher's note:** Springer Nature remains neutral with regard to jurisdictional claims in published maps and institutional affiliations.

## Supplementary Material

Supplementary Information

## Figures and Tables

**Table 1:** Grain yield and its main components in 184 chromosome segment substitution lines (CSSLs) and two test populations.

Trait	Parents			Zheng58 × lx9801		CSSL × Zheng58		
	lx9801	Zheng58	Xun9058	Mean	Mid-parent Heterosis (%)	Mean	Variance	Mid-parent Heterosis (%)
Ear length (cm)	12.19	13.98	14.55	18.03	37.79	17.89 ± 0.55	16.88–18.87	36.72
Ear width (cm)	4.24	3.96	4.2	4.7	14.63	4.66 ± 0.11	4.47–4.88	13.66
Row number	12.87	12.2	12.23	13.4	6.9	13.49 ± 0.42	12.70–14.13	7.62
Kernels per row	23.72	22.27	23.4	35.11	52.69	34.44 ± 1.34	31.23–36.92	49.77
100–kernel weight (g)	26.22	33.21	31.07	34.49	16.07	34.01 ± 1.43	30.98–36.94	14.45
Grain yield (t/ha)	6.19	6.82	7.27	11.19	72.03	11.05 ± 0.01	8.91–12.76	69.87
Trait	Chang7-2	CSSL population		Xun9058 × lx9801		CSSL × Xun9058		
	Mean	Mean	Variance	Mean	Mid-parent Heterosis (%)	Mean	Variance	Mid-parent Heterosis (%)
Ear length (cm)	10.39	12.04 ± 0.23	8.64–15.85	17.58	31.49	17.45 ± 0.67	16.13–18.50	30.52
Ear width (cm)	4.52	4.16 ± 0.06	3.79–4.64	4.87	15.4	4.74 ± 0.10	4.56–4.96	12.32
Row number	16.58	12.7 ± 0.35	11.72–14.30	13.24	5.47	13.63 ± 0.48	12.84–14.34	8.61
Kernels per row	24.56	23.55 ± 0.26	16.68–28.98	34.35	45.78	34.15 ± 1.47	31.08–36.78	44.93
100–kernel weight (g)	24.6	25.76 ± 0.19	20.21–32.47	33.87	18.23	33.49 ± 1.49	30.01–36.63	16.9
Grain yield (t/ha)	6.08	6.24 ± 0.02	3.58–9.32	11.11	65	10.97 ± 0.01	9.38–12.35	62.92

**Table 2:** Grain yield and its main components in 184 chromosome segment substitution lines (CSSLs) and two test populations.

Trait	Ear length	Ear width	Row number	Kernels per row	100-kernel weight	Grain yield
Location	40.67**	12.80**	13.98**	57.24**	63.82**	114.64**
Genetic	1.98**	1.86**	1.33*	1.45*	1.34*	1.48*
Location × Genetic	1.41*	1.25	1.07	1.10	1.04	1.01
Heritability (*H*_*B*_^*2*^)	63.02%	67.26%	68.06%	62.53%	62.08%	73.28%

Note: ^*^ and ^**^ indicate significant differences at *P* < 0.05 and *P* < 0.01, respectively.

**Table 3:** Quantitative trait loci (QTLs) detected for grain yield and its components in a chromosome segment substitution line population.

Trait	QTL	bin	Chromosomal region	*p* value	Additive	Contribution (%)
Ear length	*qEL1a*	1.03	umc1397-bnlg182-bnlg2238	6.17E-03	−1.49	−12.26
	*qEL1b*	1.08	umc1278-umc1013-bnlg2228	2.10E-02	−0.64	−5.29
	*qEL2*	2.04	bnlg1064-umc1024-umc1465	2.39E-02	0.76	6.20
	*qEL3*	3.07	umc1148-umc1489-umc1825	2.33E-02	−1.29	−10.62
	*qEL4*	4.10	umc1101-bnlg589-umc1109	4.63E-02	−0.90	−7.41
	*qEL9*	9.02	umc1170-umc1037-umc1033	6.58E-03	−1.39	−11.44
Ear width	*qEW1a*	1.05	umc2230-umc1297-umc1601	3.75E-02	−0.18	−4.33
	*qEW1b*	1.08	umc1278-umc1013-bnlg2228	4.26E-02	−0.14	−3.19
	*qEW1c*	1.11	umc2047-umc1538-bnlg131	1.65E-02	−0.15	−3.50
	*qEW4*	4.01	umc1017-umc1757-umc2280	4.33E-02	−0.08	−1.76
	*qEW5*	5.00	umc1496-umc1097-bnlg1006	1.52E-02	0.17	3.97
	*qEW6a*	6.04	umc1979-nc009-umc1014	3.81E-03	−0.15	−3.50
	*qEW6b*	6.04	mmc0523-umc2006-umc1614	2.18E-03	−0.11	−2.48
	*qEW6c*	6.08	phi123-umc1127	1.12E-02	−0.22	−5.22
	*qEW9*	9.01	bnlg1272-bnlg1810-umc1809	2.52E-03	−0.43	−10.17
Row number	*qRN2*	2.04	bnlg1064-umc1024-umc1465	2.85E-02	−0.58	−4.53
	*qRN3*	3.08	umc1844-umc2275-umc2081	2.67E-02	0.62	4.79
	*qRN4*	4.01	umc1017-umc1757-umc2280	4.09E-02	−0.36	−2.78
	*qRN5*	5.00	umc1496-umc1097-bnlg1006	2.03E-02	0.79	6.15
	*qRN6a*	6.04	umc1979-nc009-umc1014	3.27E-02	−0.38	−2.98
	*qRN6b*	6.06	bnlg1732-umc1424-umc1296	4.95E-02	−0.43	−3.37
	*qRN6c*	6.07	umc1653-umc2059-phi123	4.54E-02	−0.35	−2.72
	*qRN9*	9.05	umc1492-umc1519-umc1375	2.28E-02	0.43	3.34
Kernels per row	*qKPR1a*	1.05	umc2230-umc1297-umc1601	3.43E-04	−3.53	−14.86
	*qKPR1b*	1.06	umc1035-umc1335-umc2396	1.92E-02	2.63	11.08
	*qKPR1c*	1.08	umc1278-umc1013-bnlg2228	7.02E-03	−1.45	−6.12
	*qKPR2*	2.04	bnlg1064-umc1024-umc1465	3.26E-02	1.79	7.55
	*qKPR3*	3.08	umc1844-umc2275-umc2081	1.71E-02	3.90	16.46
	*qKPR4*	4.01	umc1017-umc1757-umc2280	3.90E-02	−2.08	−8.77
	*qKPR5*	5.00	umc1496-umc1097-bnlg1006	2.25E-02	−1.10	−4.62
	*qKPR6*	6.04	umc2006-umc1614-umc2141	1.75E-02	2.10	8.87
	*qKPR7*	7.04	umc2332-phi328175-umc1295	5.44E-03	−1.63	−6.85
100-kernel weight	*qKW1a*	1.08	umc1278-umc1013-bnlg2228	1.49E-02	−3.01	−11.47
	*qKW1b*	1.11	umc2047-umc1538-bnlg131	1.22E-02	−0.93	−3.55
	*qKW2*	2.04	bnlg1064-umc1024-umc1465	2.62E-02	3.12	11.91
	*qKW3*	3.03	phi374118-umc2258-bnlg1447	9.21E-05	−1.58	−6.02
	*qKW6a*	6.04	mmc0523-umc2006-umc1614	4.34E-02	0.52	1.99
	*qKW6b*	6.08	phi123-umc1127	1.42E-03	−1.80	−6.87
	*qKW9*	9.02	umc1170-umc1037-umc1033	9.41E-04	−1.57	−5.97
Grain yield	*qGY1*	1.05	umc2230-umc1297-umc1601	1.22E-02	−1.22	−20.02
	*qGY2*	2.04	bnlg1064-umc1024-umc1465	2.75E-03	1.01	16.86
	*qGY3*	3.08	umc1844-umc2275-umc2081	1.52E-03	0.68	11.41
	*qGY6a*	6.03	umc1178-phi389203-umc2316	8.98E-03	0.47	7.44
	*qGY6b*	6.05	mmc0523-umc2006-umc1614	7.67E-03	0.14	2.20
	*qGY9*	9.01	bnlg1272-bnlg1810-umc1809	2.14E-02	−0.88	−14.12

**Table 4:** Heterotic loci (HL) detected for grain yield and its components in a CSSL × Zheng58 population.

Traits	HL	bin	Chromosomal region	*P* value	Control heterosis(%)
Ear length	*hlEL1b*	1.04	bnlg182-bnlg2238-umc1144	7.77E-03	−4.72
	*hlEL2b*	2.04	bnlg1064-umc1024-umc1465	3.09E-03	−3.84
	*hlEL3a*	3.03	phi374118-umc2258-bnlg1447	3.96E-03	4.19
	*hlEL3b*	3.04	umc2259-phi036-umc1495	2.12E-02	3.67
	*hlEL3d*	3.07	umc1148-umc1489-umc1825	4.99E-02	−2.55
	*hlEL3e*	3.08	umc1844-umc2275-umc2081	1.49E-02	−2.76
	*hlEL6c*	6.05	umc1614-umc2141-umc1805	4.04E-03	−4.25
	*hlEL6d*	6.06	bnlg1732-umc1424-umc1296	2.00E-02	8.00
	*hlEL7a*	7.02	umc1433-bnlg1380-bnlg1792	3.41E-02	−9.10
	*hlEL7d*	7.03	bnlg2271-umc1112-bnlg1805	3.28E-02	−2.34
	*hlEL7e*	7.04	umc2332-phi328175-umc1295	3.29E-03	−6.90
	*hlEL8a*	8.03	bnlg2082-umc1741-umc2354	1.74E-04	4.99
	*hlEL8b*	8.08	umc2354-phi015-dupssr14	3.53E-02	9.94
	*hlEL9a*	9.01	bnlg1272-bnlg1810-umc1809	3.09E-02	−5.15
	*hlEL9d*	9.07	dupssr29-bnlg128-umc1982	1.96E-02	−5.71
	*hlEL10a*	10.04	umc1291-umc2163-umc2350	2.28E-02	−5.42
Ear width	*hlEW1a*	1.02	umc2191-bnlg1007-bnlg1083	3.78E-02	−2.47
	*hlEW1c*	1.05	umc2230-umc1297-umc1601	3.88E-03	−4.74
	*hlEW2b*	2.04	umc1024-umc1465-umc1541	1.06E-02	8.50
	*hlEW3b*	3.04	umc1717-umc1025-mmc0132	1.51E-03	−5.72
	*hlEW3c*	3.06	umc1593-umc1027-umc2268	4.18E-02	3.32
	*hlEW3d*	3.07	umc1148-umc1489-umc1825	1.96E-02	3.33
	*hlEW3e*	3.08	umc1844-umc2275-umc2081	2.21E-02	−2.37
	*hlEW4b*	4.10	umc1101-bnlg589-umc1109	1.94E-02	3.18
	*hlEW5a*	5.01	bnlg1006-phi024-bnlg1879	3.54E-03	−4.28
	*hlEW6a*	6.00	phi075-bnlg238-umc2309	2.33E-03	−4.14
	*hlEW6b*	6.04	mmc0523-umc2006-umc1614	3.52E-02	−3.08
	*hlEW6c*	6.06	bnlg1732-umc1424-umc1296	1.80E-02	−2.90
	*hlEW7a*	7.02	umc1666-umc1703-umc1433	3.56E-02	−4.52
	*hlEW7b*	7.03	umc1567-bnlg1305-bnlg2271	1.50E-02	4.04
	*hlEW8a*	8.02	bnlg2235-umc2004-umc1872	1.54E-02	3.11
	*hlEW8b*	8.09	dupssr14-phi233376	4.23E-03	−4.89
	*hlEW9a*	9.01	bnlg1272-bnlg1810-umc1809	3.47E-02	−5.72
	*hlEW9c*	9.05	umc1492-umc1519-umc1375	2.14E-04	5.05
	*hlEW9d*	9.07	dupssr29-bnlg128-umc1982	4.55E-02	−4.67
Row number	*hlRN1a*	1.04	bnlg182-bnlg2238-umc1144	4.84E-04	5.88
	*hlRN2b*	2.04	bnlg1064-umc1024-umc1465	3.90E-02	−3.88
	*hlRN3b*	3.04	umc1908-umc1773-phi053	1.78E-02	4.75
	*hlRN3c*	3.05	umc1174-bnlg1035-umc2127	3.52E-02	−3.40
	*hlRN3e*	3.08	umc1844-umc2275-umc2081	4.49E-03	−5.89
	*hlRN4*	4.01	umc1017-umc1757-umc2280	3.86E-02	−4.15
	*hlRN5b*	5.04	umc2302-umc1990-umc1482	2.29E-02	6.78
	*hlRN6a*	6.00	phi075-bnlg238	7.36E-03	−5.91
	*hlRN6b*	6.04	mmc0523-umc2006-umc1614	2.14E-02	−3.88
	*hlRN6c*	6.05	umc2141-umc1805-nc012	1.78E-02	4.75
	*hlRN7*	7.02	umc1703-umc1433-bnlg1380	3.48E-02	−6.62
	*hlRN9a*	9.00	bnlg1272-bnlg1810	3.74E-03	6.86
	*hlRN9b*	9.01	bnlg1810-umc1809-umc2093	2.74E-02	9.83
	*hlRN9e*	9.07	dupssr29-bnlg128-umc1982	3.52E-02	5.25
	*hlRN10*	10.04	umc1291-umc2163-umc2350	2.41E-02	−4.85
Kernels per row	*hlKPR1a*	1.02	bnlg1007-bnlg1083-umc1403	5.72E-03	−10.24
	*hlKPR1d*	1.08	umc1278-umc1013-bnlg2228	4.86E-03	10.24
	*hlKPR2a*	2.03	umc2195-umc1555-bnlg1064	4.92E-03	−11.54
	*hlKPR3c*	3.07	umc1489-umc1825-phi046	4.11E-02	5.25
	*hlKPR4a*	4.01	umc1017-umc1757-umc2280	1.18E-02	9.04
	*hlKPR4b*	4.03	umc2280-umc1550-umc2211	9.84E-03	7.28
	*hlKPR5c*	5.09	umc1792-umc1153	3.62E-02	−4.80
	*hlKPR6c*	6.06	bnlg1732-umc1424-umc1296	3.71E-03	−1.72
	*hlKPR6d*	6.08	phi123-umc1127	9.58E-03	7.68
	*hlKPR7a*	7.02	umc1695-umc1666-umc1703	8.01E-03	12.99
	*hlKPR7c*	7.04	umc2332-phi328175-umc1295	2.03E-03	−3.90
	*hlKPR8*	8.09	dupssr14-phi233376	2.68E-02	−7.63
	*hlKPR9a*	9.01	bnlg1272-bnlg1810-umc1809	1.80E-02	7.81
	*hlKPR9c*	9.05	umc1492-umc1519-umc1375	7.49E-03	−4.15
	*hlKPR10a*	10.04	umc1291-umc2163-umc2350	2.64E-02	−11.05
100-kernel weight	*hlKW1a*	1.02	umc2191-bnlg1007-bnlg1083	2.12E-02	−7.94
	*hlKW1d*	1.08	bnlg2228-dupssr12-umc2047	2.19E-02	3.74
	*hlKW2a*	2.04	bnlg1064-umc1024-umc1465	2.98E-02	−5.84
	*hlKW3a*	3.04	umc2259-phi036-umc1495	5.97E-04	7.12
	*hlKW3b*	3.05	umc1954-umc2166-umc1593	1.88E-02	11.13
	*hlKW3d*	3.07	umc1148-umc1489-umc1825	3.21E-02	−6.40
	*hlKW3f*	3.08	umc1844-umc2275-umc2081	2.37E-02	12.60
	*hlKW5c*	5.06	phi085-phi048-umc2201	2.55E-02	10.03
	*hlKW5d*	5.07	umc1729-bnlg118-umc1792	4.51E-02	7.10
	*hlKW6b*	6.03	umc1178-phi389203-umc2316	1.66E-02	11.51
	*hlKW6c*	6.04	mmc0523-umc2006-umc1614	7.41E-03	−10.17
	*hlKW6e*	6.06	bnlg1732-umc1424-umc1296	4.93E-02	6.17
	*hlKW6f*	6.07	bnlg1136-umc1653-umc2059	1.88E-02	−11.54
	*hlKW6e*	6.08	phi123-umc1127	3.67E-04	−4.91
	*hlKW7a*	7.02	umc1666-umc1703-umc1433	7.47E-03	15.82
	*hlKW7b*	7.03	umc1567-bnlg1305-bnlg2271	2.36E-02	7.51
	*hlKW9a*	9.00	bnlg1272-bnlg1810	3.62E-02	−10.37
	*hlKW9b*	9.01	bnlg1810-umc1809-umc2093	4.07E-02	6.08
	*hlKW9d*	9.03	umc1170-umc1037-umc1033	2.26E-02	9.97
Grain yield	*hlGY1a*	1.03	umc1403-umc1397-bnlg182	2.17E-02	8.88
	*hlGY1b*	1.04	bnlg182-bnlg2238-umc1144	3.85E-02	−9.30
	*hlGY1d*	1.08	bnlg2228-dupssr12-umc2047	1.79E-02	11.04
	*hlGY2b*	2.04	bnlg1064-umc1024-umc1465	7.01E-03	−4.44
	*hlGY3a*	3.03	phi374118-umc2258-bnlg1447	3.71E-02	9.48
	*hlGY3d*	3.05	umc1954-umc2166-umc1593	4.54E-02	10.77
	*hlGY4*	4.01	umc1017-umc1757-umc2280	7.52E-03	−13.24
	*hlGY5*	5.05	umc1155-bnlg278-umc1680	1.82E-02	7.24
	*hlGY6b*	6.05	umc2141-umc1805-nc012	4.54E-02	−8.68
	*hlGY6c*	6.07	bnlg1136-umc1653-umc2059	4.85E-02	−8.98
	*hlGY8*	8.09	dupssr14-phi233376	1.42E-02	−16.62
	*hlGY9a*	9.02	umc1170-umc1037-umc1033	1.63E-03	−14.45
	*hlGY9c*	9.06	umc1310-umc2207-dupssr29	1.31E-02	10.28
	*hlGY10*	10.04	umc1291-umc2163-umc2350	7.81E-03	−9.42

**Table 5:** Heterotic loci (HL) detected for grain yield and its main components in a CSSL × Xun9058 population.

Traits	HL	Bin	Chromosomal region	*P* value	Control heterosis (%)
Ear length	*hlEL1a*	1.03	umc1403-umc1397-bnlg182	1.56E-03	−15.85
	*hlEL1b*	1.04	bnlg182-bnlg2238-umc1144	1.14E-03	6.04
	*hlEL1c*	1.08	umc1278-umc1013-bnlg2228	4.58E-02	−3.04
	*hlEL2a*	2.03	umc2195-umc1555-bnlg1064	3.70E-02	−8.43
	*hlEL2b*	2.04	bnlg1064-umc1024-umc1465	4.48E-02	−1.94
	*hlEL3c*	3.05	umc1954-umc2166-umc1593	5.51E-03	6.12
	*hlEL4*	4.01	umc1017-umc1757-umc2280	2.83E-02	−6.74
	*hlEL6a*	6.03	umc1178-phi389203-umc2316	3.98E-02	5.81
	*hlEL6b*	6.05	mmc0523-umc2006-umc1614	3.45E-04	−5.33
	*hlEL6d*	6.06	bnlg1732-umc1424-umc1296	4.78E-02	−1.91
	*hlEL6e*	6.07	bnlg1136-umc1653-umc2059	1.08E-02	6.75
	*hlEL7b*	7.02	umc1695-umc1666-umc1703	3.55E-03	11.38
	*hlEL7c*	7.03	bnlg1792-umc1929-umc1585	2.79E-02	−5.43
	*hlEL7e*	7.04	umc2332-phi328175-umc1295	4.25E-02	−7.73
	*hlEL9b*	9.05	umc1492-umc1519-umc1375	1.45E-02	11.51
	*hlEL9c*	9.06	bnlg1091-bnlg1191-umc2345	4.25E-02	−8.23
	*hlEL10b*	10.04	umc2350-umc1272-umc2221	2.83E-02	−8.55
Ear width	*hlEW1b*	1.03	umc1403-umc1397-bnlg182	7.58E-03	−4.87
	*hlEW1c*	1.05	umc2230-umc1297-umc1601	3.40E-02	−2.53
	*hlEW1d*	1.11	umc2047-umc1538-bnlg131	2.96E-02	−2.75
	*hlEW2a*	2.04	bnlg1064-umc1024-umc1465	1.67E-02	−2.86
	*hlEW2c*	2.08	umc1806-umc2202-umc1516	4.66E-02	6.11
	*hlEW3a*	3.03	phi374118-umc2258-bnlg1447	3.59E-02	−6.54
	*hlEW4a*	4.01	umc1017-umc1757-umc2280	3.34E-02	−4.27
	*hlEW5b*	5.06	phi048-umc2201-bnlg1306	4.85E-02	3.48
	*hlEW6a*	6.00	phi075-bnlg238-umc2309	2.64E-02	−4.17
	*hlEW6b*	6.04	mmc0523-umc2006-umc1614	3.49E-02	−8.83
	*hlEW6c*	6.06	bnlg1732-umc1424-umc1296	3.63E-02	−4.37
	*hlEW6d*	6.07	bnlg1136-umc1653-umc2059	4.46E-02	−3.90
	*hlEW7c*	7.04	umc2332-phi328175-umc1295	5.77E-03	−4.28
	*hlEW9b*	9.02	umc1170-umc1037-umc1033	1.87E-03	−6.29
Row number	*hlRN1a*	1.04	bnlg182-bnlg2238-umc1144	4.18E-02	7.92
	*hlRN1b*	1.08	umc1278-umc1013-bnlg2228	1.82E-03	5.78
	*hlRN2a*	2.02	umc2403-umc1265-umc1961	3.79E-02	8.46
	*hlRN3a*	3.04	umc2259-phi036-umc1495	1.93E-02	7.80
	*hlRN3d*	3.07	umc1489-umc1825-phi046	1.57E-02	2.68
	*hlRN4*	4.01	umc1017-umc1757-umc2280	8.36E-04	8.39
	*hlRN5a*	5.00	umc1496-umc1097-bnlg1006	3.32E-02	6.12
	*hlRN5c*	5.06	bnlg278-umc1680-phi085	7.97E-03	6.47
	*hlRN6d*	6.07	bnlg1136-umc1653-umc2059	3.21E-02	−5.97
	*hlRN8*	8.02	bnlg2235-umc2004-umc1872	7.97E-03	6.47
	*hlRN9a*	9.00	bnlg1272-bnlg1810	1.23E-02	4.95
	*hlRN9c*	9.02	umc1170-umc1037-umc1033	3.38E-03	4.61
	*hlRN9d*	9.05	umc1492-umc1519-umc1375	4.24E-02	4.29
	*hlRN9e*	9.07	dupssr29-bnlg128-umc1982	2.57E-02	−3.96
	*hlRN10*	10.04	umc1291-umc2163-umc2350	3.65E-02	4.69
Kernels per row	*hlKPR1a*	1.02	bnlg1007-bnlg1083-umc1403	1.66E-02	8.41
	*hlKPR1b*	1.04	bnlg182-bnlg2238-umc1144	3.75E-02	4.46
	*hlKPR1c*	1.05	umc2230-umc1297-umc1601	4.24E-02	−9.52
	*hlKPR1e*	1.11	umc2047-umc1538-bnlg131	4.90E-02	5.45
	*hlKPR2a*	2.03	umc2195-umc1555-bnlg1064	1.06E-03	7.95
	*hlKPR2b*	2.04	bnlg1064-umc1024-umc1465	1.82E-02	−7.77
	*hlKPR3a*	3.04	umc1717-umc1025-mmc0132	1.39E-02	−10.01
	*hlKPR3b*	3.05	umc1174-bnlg1035-umc2127	8.01E-03	−3.50
	*hlKPR3d*	3.08	umc1844-umc2275-umc2081	1.36E-02	4.17
	*hlKPR5a*	5.04	umc2302-umc1990-umc1482	2.73E-02	−6.64
	*hlKPR5b*	5.06	phi085-phi048-umc2201	4.41E-02	−6.59
	*hlKPR6a*	6.00	phi075-bnlg238-umc2309	7.47E-03	8.53
	*hlKPR6b*	6.05	umc1614-umc2141-umc1805	8.45E-03	5.94
	*hlKPR7a*	7.02	umc1695-umc1666-umc1703	1.78E-03	8.53
	*hlKPR7b*	7.03	bnlg2271-umc1112-bnlg1805	1.10E-02	7.95
	*hlKPR9b*	9.03	umc1170-umc1037-umc1033	3.44E-02	−5.57
	*hlKPR9d*	9.05	umc1231-umc1494-bnlg1091	2.74E-02	−8.29
	*hlKPR10b*	10.04	umc2350-umc1272-umc2221	2.96E-04	−12.79
100-kernel weight	*hlKW1b*	1.03	umc1403-umc1397-bnlg182	4.23E-02	−9.11
	*hlKW1c*	1.04	bnlg182-bnlg2238-umc1144	4.16E-02	15.35
	*hlKW2b*	2.04	umc2088-umc1485-bnlg1861	3.60E-02	−8.02
	*hlKW3b*	3.05	umc1954-umc2166-umc1593	4.97E-02	11.59
	*hlKW3c*	3.06	umc1593-umc1027-umc2268	4.99E-02	−7.82
	*hlKW3e*	3.07	umc1489-umc1825-phi046	4.99E-02	−7.82
	*hlKW4*	4.01	umc1017-umc1757-umc2280	2.34E-02	−7.53
	*hlKW5a*	5.01	bnlg1006-phi024-bnlg1879	3.01E-04	6.74
	*hlKW5b*	5.05	umc1155-bnlg278-umc1680	1.71E-03	5.98
	*hlKW6a*	6.00	phi075-bnlg238-umc2309	8.10E-03	−14.65
	*hlKW6d*	6.05	umc1614-umc2141-umc1805	4.73E-02	5.77
	*hlKW6e*	6.08	phi123-umc1127	1.91E-02	−14.90
	*hlKW7a*	7.02	umc1666-umc1703-umc1433	4.08E-02	−12.69
	*hlKW8*	8.03	bnlg1194-umc2352-bnlg2235	1.52E-05	6.74
	*hlKW9a*	9.00	bnlg1272-bnlg1810	3.56E-02	−12.60
	*hlKW9b*	9.01	bnlg1810-umc1809-umc2093	6.38E-03	−14.19
	*hlKW9c*	9.02	umc1170-umc1037-umc1033	2.92E-02	−8.33
Grain yield	*hlGY1a*	1.03	umc1403-umc1397-bnlg182	6.40E-03	−8.63
	*hlGY1c*	1.05	umc2230-umc1297-umc1601	4.91E-02	−12.99
	*hlGY1d*	1.08	bnlg2228-dupssr12-umc2047	4.79E-02	11.42
	*hlGY2a*	2.04	umc1024-umc1465-umc1541	2.12E-03	9.18
	*hlGY2c*	2.08	umc1806-umc2202-umc1516	1.85E-02	11.18
	*hlGY3a*	3.03	phi374118-umc2258-bnlg1447	9.34E-03	−17.24
	*hlGY3b*	3.04	umc1908-umc1773-phi053	9.48E-04	12.08
	*hlGY3c*	3.05	bnlg1035-umc2127-umc1954	4.81E-03	−9.19
	*hlGY3e*	3.07	umc1489-umc1825-phi046	3.95E-02	5.50
	*hlGY3f*	3.08	umc1844-umc2275-umc2081	7.10E-03	7.57
	*hlGY6a*	6.00	phi075-bnlg238-umc2309	4.41E-03	7.80
	*hlGY6c*	6.07	bnlg1136-umc1653-umc2059	6.11E-03	18.00
	*hlGY7a*	7.02	umc1666-umc1703-umc1433	3.44E-02	12.08
	*hlGY7b*	7.04	umc2332-phi328175-umc1295	2.75E-03	−18.46
	*hlGY9b*	9.02	umc1037-umc1033-bnlg1082	4.39E-02	11.25
	*hlGY9d*	9.07	dupssr29-bnlg128-umc1982	1.32E-02	−6.60

**Table 6:** QTL and HL located on the same chromosomal region detected in the CSSLs and two test populations.

Trait	CSSLs			CSSL × Zheng58		CSSL × Xun9058	
	QTL	Additive	Contribution (%)	HL	Control heterosis (%)	HL	Control heterosis (%)
Ear length	*qEL1a*	−1.49	−12.26			*hlEL1a*	−15.85
				*hlEL1b*	−4.72	*hlEL1b*	6.04
	*qEL1b*	−0.64	−5.29			*hlEL1c*	−3.04
	*qEL2*	0.76	6.20	*hlEL2b*	−3.84	*hlEL2b*	−1.94
	*qEL3*	−1.29	−10.62	*hlEL3d*	−2.55		
				*hlEL6d*	8.00	*hlEL6d*	−1.91
				*hlEL7e*	−6.90	*hlEL7e*	−7.73
Ear width	*qEW1a*	−0.18	−4.33	*hlEW1c*	−4.74	*hlEW1c*	−2.53
	*qEW1c*	−0.15	−3.50			*hlEW1d*	−2.75
	*qEW4*	−0.08	−1.76			*hlEW4a*	−4.27
				*hlEW6a*	−4.14	*hlEW6a*	−4.17
	*qEW6b*	−0.11	−2.48	*hlEW6b*	−3.08	*hlEW6b*	−8.83
				*hlEW6c*	−2.90	*hlEW6c*	−4.37
	*qEW9*	−0.43	−10.17	*hlEW9a*	−5.72		
Row number				*hlRN1a*	5.88	*hlRN1a*	7.92
	*qRN2*	−0.58	−4.53	*hlRN2b*	−3.88		
	*qRN3*	0.62	4.79	*hlRN3e*	−5.89		
	*qRN4*	−0.36	−2.78	*hlRN4*	−4.15	*hlRN4*	8.39
	*qRN5*	0.79	6.15			*hlRN5a*	6.12
				*hlRN9a*	6.86	*hlRN9a*	4.95
	*qRN9*	0.43	3.34			*hlRN9d*	4.29
				*hlRN9e*	5.25	*hlRN9e*	−3.96
				*hlRN10*	−4.85	*hlRN10*	4.69
				*hlKPR1a*	−10.24	*hlKPR1a*	8.41
Kernels per row	*qKPR1a*	−3.53	−14.86			*hlKPR1c*	−9.52
	*qKPR1c*	−1.45	−6.12	*hlKPR1d*	10.24		
				*hlKPR2a*	−11.54	*hlKPR2a*	7.95
	*qKPR2*	1.79	7.55			*hlKPR2b*	−7.77
	*qKPR3*	3.90	16.46			*hlKPR3d*	4.17
	*qKPR4*	−2.08	−8.77	*hlKPR4a*	9.04		
				*hlKPR7a*	12.99	*hlKPR7a*	8.53
	*qKPR7*	−1.63	−6.85	*hlKPR7c*	−3.90		
100-kernel weight	*qKW2*	3.12	11.91	*hlKW2a*	−5.84		
				*hlKW3b*	11.13	*hlKW3b*	11.59
	*qKW6a*	0.52	1.99	*hlKW6c*	−10.17		
	*qKW6b*	−1.80	−6.87	*hlKW6e*	−4.91	*hlKW6g*	−14.90
				*hlKW7a*	15.82	*hlKW7a*	−12.69
				*hlKW9a*	−10.37	*hlKW9a*	−12.60
				*hlKW9b*	6.08	*hlKW9b*	−14.19
	*qKW9*	−1.57	−5.97			*hlKW9c*	−8.33
				*hlGY1a*	8.88	*hlGY1a*	−8.63
Grain yield	*qGY1*	−1.22	−20.02			*hlGY1c*	−12.99
				*hlGY1d*	11.04	*hlGY1d*	11.42
	*qGY2*	1.01	16.86	*hlGY2b*	−4.44		
				*hlGY3a*	9.48	*hlGY3a*	−17.24
	*qGY3*	0.68	11.41			*hlGY3f*	7.57
				*hlGY6c*	−8.98	*hlGY6c*	18.00
