# Supplementary material for: Identification of heterotic loci associated with grain yield and its components using two CSSL test populations in maize
Source: Sci Rep. 2016 Dec 5;6:38205. doi: 10.1038/srep38205 (PMC5137037; doi:10.1038/srep38205)
Supplement: Supplementary Information [file srep38205-s1.doc]

Supplementary Information File

**Identification of heterotic loci associated with grain yield and its components using two CSSL test populations in maize**

Hongqiu Wang, Xiangge Zhang, Huili Yang, Xiaoyang Liu, Huimin Li, Liang Yuan, Weihua Li, Zhiyuan Fu, Jihua Tang & Dingming Kang

**
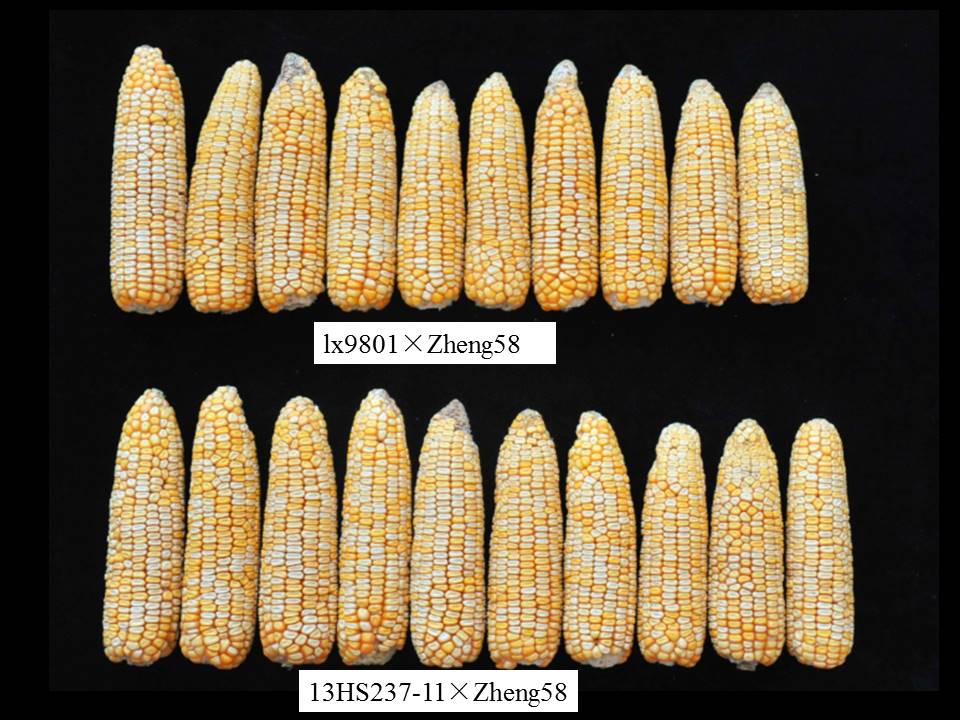
**

**a**

**b**

**Supplementary Figure 1. Ear width of the lx9801 × Zheng58 hybrid and the sub-CSSL test hybrid (13HS237-11 × Zheng58).** (a) The lx9801 × Zheng58 hybrid. (b) The sub-CSSL test hybrid.


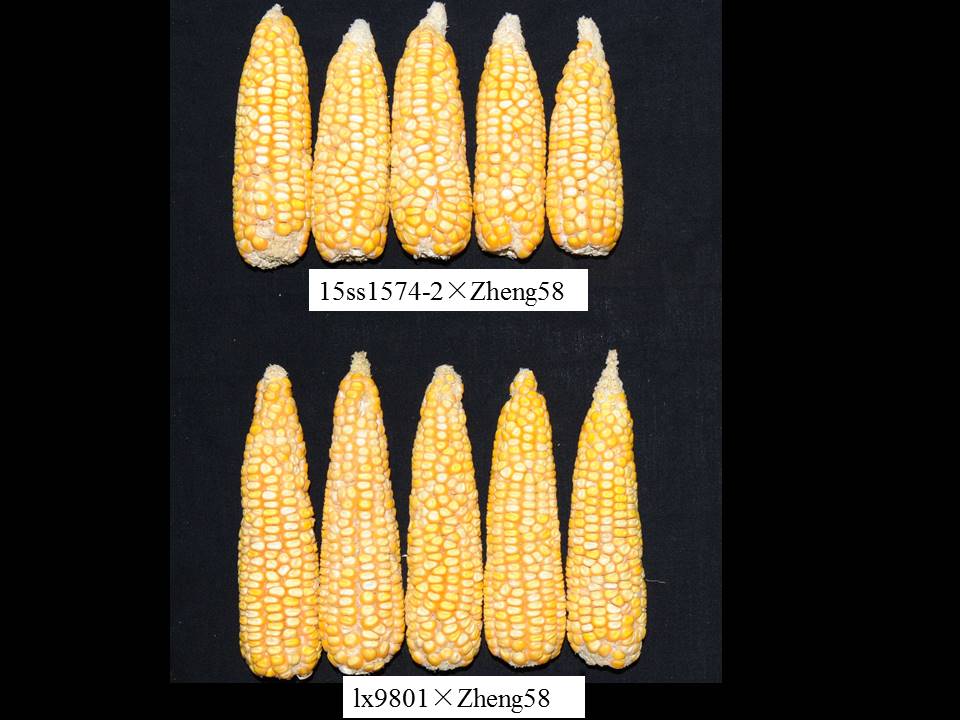


**a**

**b**

**Supplementary Figure 2. Ear length of the sub-CSSL test hybrid (15SS1574-2 × Zheng58) and** **the lx9801 × Zheng58 hybrid.** (a) The sub-CSSL test hybrid. (b) The lx9801 × Zheng58 hybrid.

**Supplementary Table 1. Sub-CSSLs harbouring the heterotic locus *hlEW2b* detected for ear width in a Zheng58×CSSL population.**

| Location | Changge(mm) | Xunxian (mm) |
| --- | --- | --- |
| lx9801×Zheng58 | 50.36±0.28 | 47.80±0.52 |
| 13HS222-1×Zheng58 | 51.46±0.24^*^ | 50.31±0.54^**^ |
| *P* value | 1.1×10**^−2^** | 3.5×10**^−3^** |
| lx9801×Zheng58 | 50.36±0.28 | 48.11±0.43 |
| 13HS237-11×Zheng58 | 52.63±0.39^**^ | 50.35±0.53^**^ |
| *P* value | 1.7×10**^−4^** | 4.1×10**^−3^** |
| lx9801×Zheng58 | 50.36±0.28 | 48.11±0.43 |
| 13HS241-1×Zheng58 | 52.19±0.38^**^ | 50.98±0.62^**^ |
| *P* value | 1.2×10**^−3^** | 1.3×10**^−3^** |

Note: ^*^ and ^**^ indicate significant differences at *P* < 0.05 and *P* < 0.01, respectively.

**Supplementary Table 2. Sub-CSSLs harbouring the heterotic locus *hlEL3d* detected for ear length in a Zheng58×CSSL population**.

| Location | Xishuangbanna | Sanya |
| --- | --- | --- |
| lx9801×Zheng58 | 21.80±0.31^**^ | 13.56±0.20^**^ |
| 15ss1574-2×Zheng58 | 18.38±0.64 | 12.0±0.13 |
| *P* value | 1.4×10**^−3^** | 4.9×10**^−4^** |
| lx9801×Zheng58 | 21.52±0.48^**^ | 14.6±0.22^**^ |
| 15ss1590-1×Zheng58 | 17.70±0.73 | 11.98±0.68 |
| *P* value | 4.8×10**^−3^** | 6.4×10**^−3^** |
| lx9801×Zheng58 | 20.88±0.23^**^ | 14.82±0.46^*^ |
| 15ss1597-2×Zheng58 | 17.60±0.71 | 12.86±0.57 |
| *P* value | 2.2×10**^−3^** | 2.9×10**^−2^** |
| lx9801×Zheng58 | 20.88±0.23^**^ | 14.82±0.46^*^ |
| 15ss1597-12×Zheng58 | 18.12±0.67 | 13.10±0.35 |
| *P* value | 4.7×10**^−3^** | 1.9×10**^−2^** |
| lx9801×Zheng58 | 21.52±0.48^**^ | 15.16±0.34^**^ |
| 15ss1582-12×Zheng58 | 17.17±0.41 | 12.16±0.50 |
| *P* value | 5×10**^−4^** | 1.1×10**^−3^** |

Note: ^*^ and ^**^ indicate significant differences at *P* < 0.05 and *P* < 0.01, respectively.
